# Multiscale Interfacial Engineering Enables Robust Reconstituted Wood

**DOI:** 10.1002/advs.78399

**Published:** 2026-10-28

**Authors:** Huizheng Wu, Yi Kong, Liangxian Liu, Yuyan Jiang, Jiahuan He, Haiyu Li, Ming Wei, Dexiu Min, Jian Li, Yanjun Xie, Chi Zhang, Shaoliang Xiao

**Affiliations:** ^1^ Key Laboratory of Bio‐based Material Science & Technology (Ministry of Education) Northeast Forestry University Harbin P. R. China; ^2^ Engineering Research Center of Advanced Wooden Materials (Ministry of Education) Northeast Forestry University Harbin P. R. China; ^3^ Shandong Xingang Enterprise Group Co., Ltd Shandong P. R. China; ^4^ School of Flexible Electronics (SoFE) Sun Yat‐sen University Shenzhen P. R. China; ^5^ State Key Laboratory of Optoelectronic Materials and Technologies (OEMT) Sun Yat‐sen University Guangzhou P. R. China

**Keywords:** interfacial welding, ionic liquids, lightweight and high‐strength, reconstituted wood

## Abstract

Development of sustainable, high‐performance structural materials capable of replacing traditional engineering plastics and lightweight alloys is critical to achieving carbon neutrality. However, engineering renewable wood into structural materials intrinsically relies on reconstitution and bonding, during which traditional polymer adhesives introduce compositionally and mechanically heterogeneous interfaces. These discontinuities produce stress concentrations under water‐induced swelling and shrinkage, undermining long‐term stability. Herein, we proposed a synergistic strategy integrating interfacial reconstruction and water‐induced regeneration to reconstruct heterogeneous interfaces into homogeneous ones with compositional and structural continuity. This approach enabled lap joints to achieve a high dry shear strength of 6.2 MPa, exceeding that of natural wood. Notably, this strategy utilized water to facilitate the regeneration and performance retention of the interface, allowing the material to retain 97% of its strength even after two boiling water cycles. Furthermore, the mechanical properties were optimized through pressure control, thereby yielding specific flexural strength (276.2 MPa cm^3^ g^−1^) and specific flexural modulus (24 000 MPa cm^3^ g^−1^) that were comparable or even superior to those of certain metals, construction materials, and engineering plastics. This work provides useful insights into the design of high‐performance, sustainable wood‐based structural materials suitable for outdoor environments.

## Graphical abstract

Conventional adhesives used in wood composites create heterogeneous interfaces that cause stress concentration under humid or aqueous conditions, thereby compromising long‐term stability. A synergistic strategy integrating interfacial reconstruction and water‐induced regeneration transforms heterogeneous interfaces into homogeneous ones with compositional and structural continuity. The resulting sustainable reconstituted structural material exhibits high strength, excellent water resistance, and tunable performance.

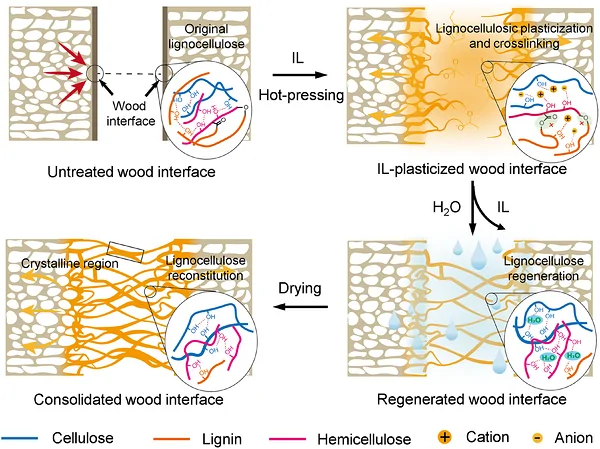

## 1 Introduction

Lightweight, high‐strength, and sustainable structural materials are crucial for aerospace, transportation, and construction applications [1, 2, 3]. However, conventional structural materials, such as metals, ceramics, and engineering plastics, require energy‐intensive manufacturing processes, and they have been associated with high energy consumption and carbon emissions, posing challenges in achieving global carbon neutrality [4, 5, 6, 7]. Therefore, developing sustainable alternative materials that offer both exceptional mechanical properties and a low environmental footprint is imperative. This helps in mitigating the energy and environmental costs associated with using conventional plastics and metals [8, 9, 10].

Natural wood, owing to its low cost and excellent mechanical performance, has been widely used as a structural material for constructing buildings and furniture for thousands of years [11, 12, 13, 14]. The development of wood materials with tailored performance has attracted extensive attention, driven by a deepening understanding of structure‐property relationships [15, 16, 17, 18]. For instance, strategies such as selective component removal combined with densification and polymer impregnation, have been employed to significantly enhance the bulk mechanical properties of wood [13, 19]. However, engineering wood into large‐scale structural materials typically requires reconstitution and bonding processes [20]. Currently, achieving an interfacial bonding strength higher than that of natural wood, together with excellent water resistance, remains a major challenge. An important reason for this is associated with the intrinsic heterogeneous interfaces of conventional adhesive systems. Specifically, regardless of whether petrochemical‐based (e.g., urea‐formaldehyde [UF] and phenol‐formaldehyde [PF]) or bio‐based adhesives are utilized, these systems exhibit mismatches with the wood matrix in both composition and chemical structure [21, 22, 23, 24, 25, 26]. These mismatches induce substantial volumetric and thermal stresses during the bonding process, thereby producing stress concentration sites at the heterogeneous interfaces. The environmental disturbances (particularly moisture) during material service cause uncoordinated swelling and shrinkage between the wood matrix and the adhesive [27]. This not only increases stress concentrations at the interfaces but also accelerates the hydrolysis of interfacial chemical bonds. These stress concentration points cause the initiation and propagation of cracks at these locations, ultimately leading to bond failure. Therefore, developing a strategy that can mitigate heterogeneous interfacial defects, while imparting high strength and exceptional water resistance to the bonded interface, is crucial. Such a strategy would also enable tunable performance of the reconstituted structural materials, thereby promoting bio‐based materials toward advanced engineering applications.

This study proposes a synergistic strategy that combines interfacial reconstruction with water‐induced regeneration to create a seamless interlocking interface with high strength and excellent water resistance. An ionic liquid (IL) is used for in situ plasticization and molecular welding of the cell walls, effectively reducing interfacial heterogeneity. Subsequently, the plasticized interface maintains its mechanical performance after water‐induced regeneration and final consolidation (Figure 1a). Moreover, by regulating the processing pressure, the fabricated IL‑plasticized wood sustainable reconstituted structural material (IPW‑SRSM) can achieve both high strength and high toughness, with comprehensive properties that are comparable or even superior to those of certain engineering plastics and aluminum (Figure 1b). This work provides a promising and sustainable pathway for designing high‑performance wood‑based structural materials suitable for high‑humidity and extreme service environments, such as those used in construction, automotive, and aerospace components (Figure 1c).

**FIGURE 1:**
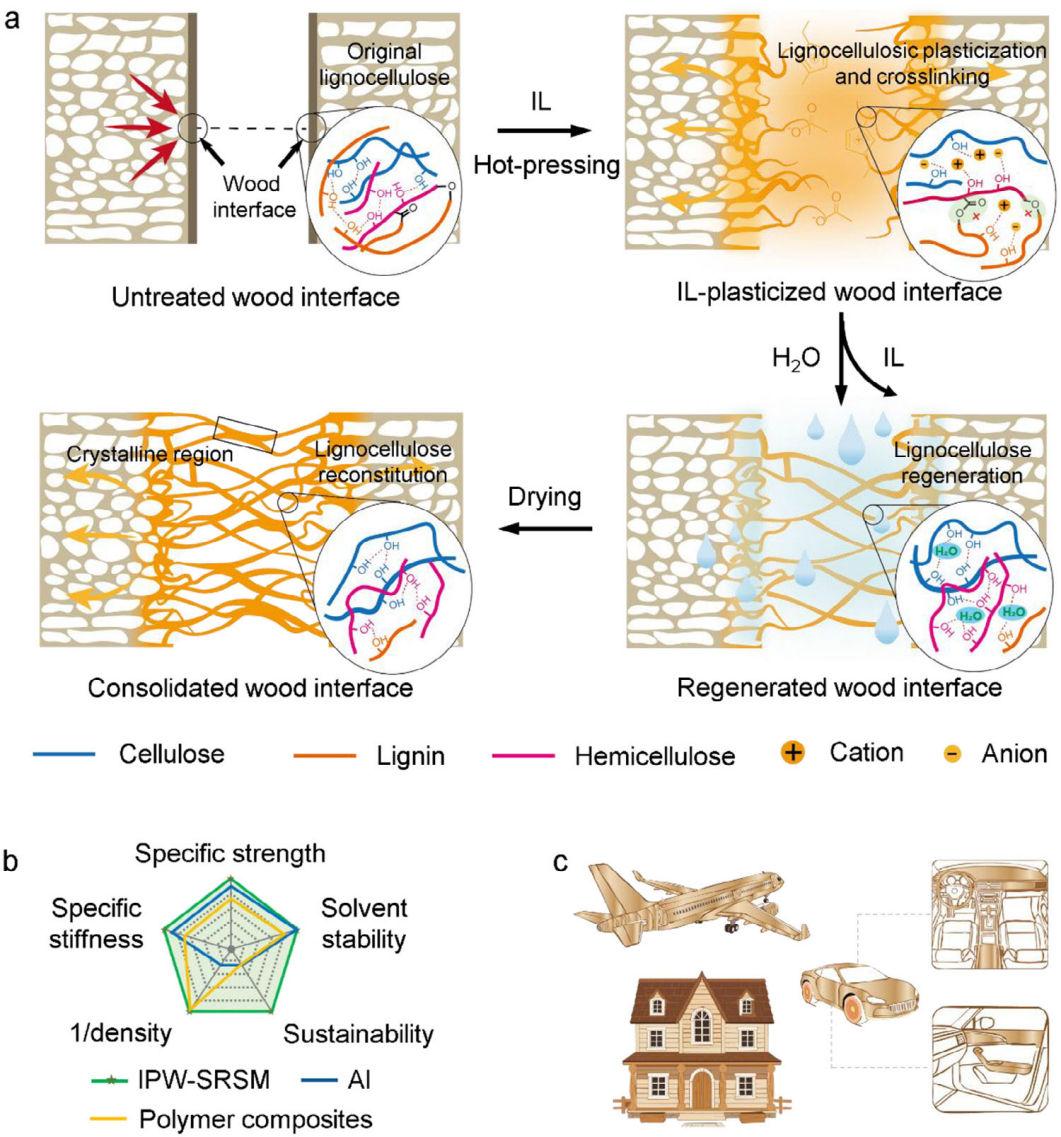
Fabrication of the IL‑plasticized wood sustainable reconstituted structural material (IPW‑SRSM) using a synergistic strategy combining interfacial reconstruction and water‑induced regeneration. (a) Schematic diagrams illustrating the mechanisms governing the untreated, IL‐plasticized, regenerated, and consolidated states of the wood interface. (b) Radar plot comparing the overall performance of IPW‐SRSM, polymer composites, and aluminum. (c) Representative examples illustrating the potential of IPW‐SRSM in high‐performance applications, including construction, automotive, and aerospace components.

## 2 Results and Discussion

### 2.1 High‐Strength Interfacial Bonding Enabled by IL Plasticization and Hot Pressing

To verify the feasibility of the proposed in situ plasticization and welding strategy for native cell walls, various ILs (1‐ethyl‐3‐methylimidazolium acetate ([Emim]Ac), 1‐allyl‐3‐methylimidazolium chloride ([Amim]Cl), 1‐ethyl‐3‐methylimidazolium diethyl phosphate ([Emim]DEP), 1,3‐dimethylimidazolium methyl phosphonate ([Mmim]MP), 1‐ethyl‐3‐methylimidazolium methyl phosphonate ([Emim]MP), and 1‐butyl‐3‐methylimidazolium methyl phosphonate ([Bmim]MP)) and wood species (poplar, pine, birch, and teak) were investigated. The adhesion performance of the welded interface was evaluated by measuring the tensile shear strength of the lap joints. As shown in Figure 2a and Figure S1a, [Emim]Ac more effectively and synergistically plasticized wood components owing to its appropriate molecular size and hydrogen bond basicity [28]. Moreover, the relatively low extractive content in poplar wood allowed the IL to penetrate and plasticize the wood matrix effectively. Consequently, [Emim]Ac exhibited optimal welding performance on poplar wood (Figure S1b), and this combination was ultimately selected as the primary system for this study.

**FIGURE 2:**
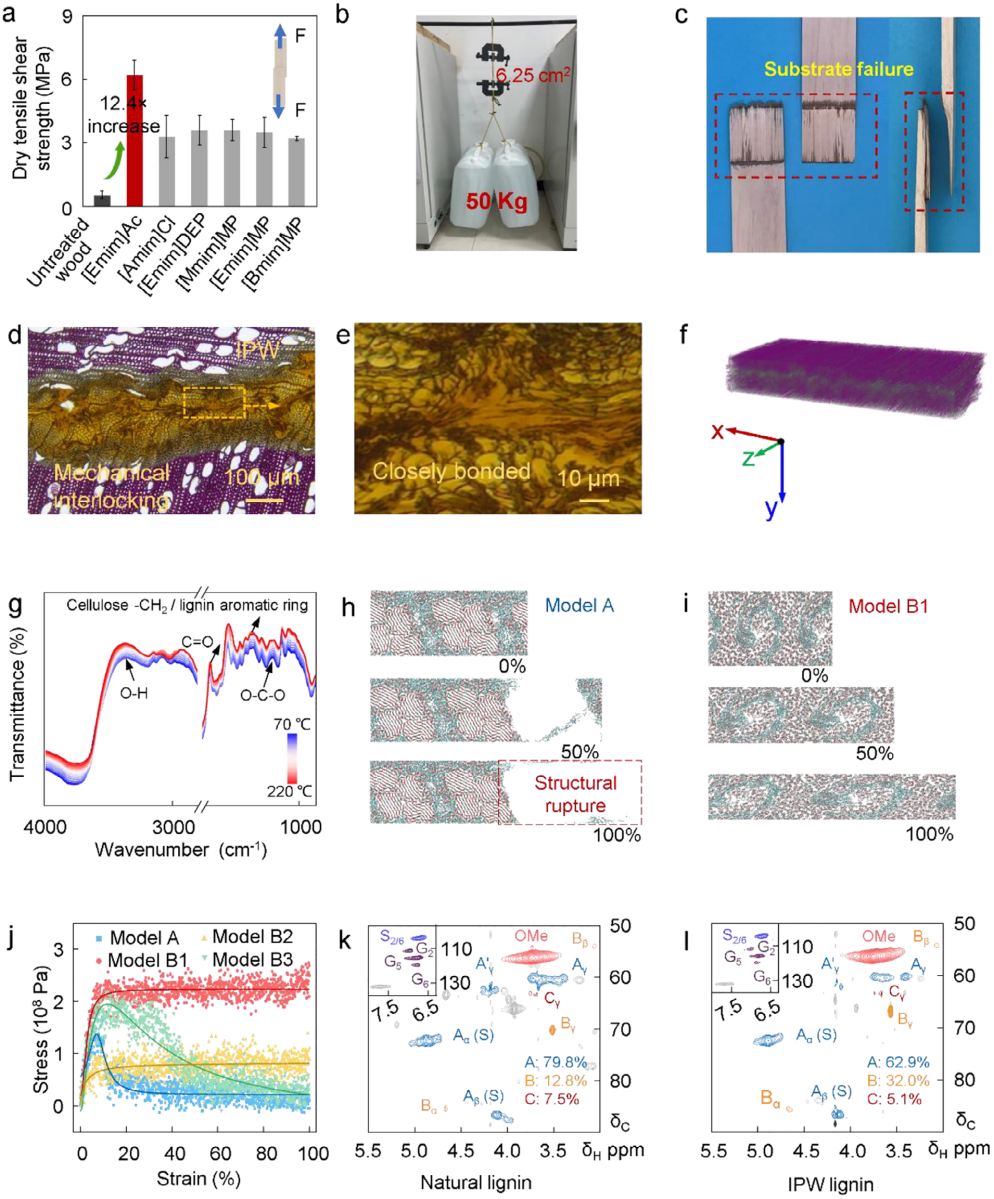
High‐strength interfacial bonding enabled by IL plasticization and hot pressing. (a) Dry tensile shear strength of untreated wood and six types of IL‑plasticized wood (IPW) lap joints. (b) Photograph of an IPW lap joint (bonding area of 6.25 cm^2^) supporting a 50 kg load. (c) Failure mode analysis of IPW lap joints. (d) Optical microscopy image revealing the mechanical interlocking structure of the IPW lap joint. (e) Magnified optical microscopy image demonstrating the exceptional compatibility between plasticized cell walls and wood matrix. (f) Micro‐computed tomography (Micro‐CT) three‐dimensional reconstruction of the IPW lap joint. (g) Temperature‐dependent FT‐IR spectra of IPW in the 70°C–220°C range. (h, i) Morphological evolution during tensile deformation for (h) Model A (untreated wood interface) and (i) Model B1 (IPW interface). (j) Stress–strain curves obtained from molecular dynamics (MD) simulations for Model A, Model B1, Model B2 (regenerated wood interface), and Model B3 (consolidated wood interface). Detailed information regarding the modeling and simulation protocols is provided in Supporting Information and in our previous report [32, 33, 34]. Two‐dimensional heteronuclear single‐quantum correlation nuclear magnetic resonance (2D HSQC NMR) spectra of side‐chain regions for (k) natural lignin and (l) IPW lignin, showing the aliphatic (δC/δH 50–90/3.0–5.5) and aromatic (δC/δH 90–140/6.0–8.0) regions [30, 35]. Key lignin structures are assigned as follows: A, β–O–4 aryl ether, B, β–5 phenylcoumaran, C, β–β resinol, S, syringyl, and G, guaiacyl.

Following hot pressing at 180°C and 3 MPa for 30 min with an IL mass of 0.75 g (Figure S2), the fabricated IL‑plasticized wood (IPW) lap joints exhibited an exceptional dry shear strength of 6.2 MPa, a 12.4‐fold increase compared with that of untreated wood (0.5 MPa). Macroscopically, a joint with a welded area of merely 6.25 cm^2^ supported a 50 kg load (Figure 2b). An analysis of the failure mode of the joint indicated that the fracture occurred entirely within the wood matrix rather than at the interface, with a wood failure rate of close to 100%, demonstrating high load‐bearing capacity (Figure 2c). This macroscopic evidence proved that the interfacial strength achieved via IL plasticization welding surpassed the bulk strength of natural wood, demonstrating that the interface was no longer the weak region of the material.

This high‐strength interfacial bonding mainly originated from a cross‐scale synergistic strengthening mechanism triggered by IL plasticization and hot pressing. Specifically, this synergistic enhancement occurred at three distinct levels, as described below.

#### 2.1.1 Microscale Mechanical Interlocking and Physical Densification

The IL effectively penetrated and disrupted the rigid structure of the cell wall. Processing at 180°C for 17 min caused the microscopic pore structure of the cell wall to undergo plastic collapse and transform into a deformable viscoelastic state (Figure S3a–e and Videos S1–S3). This in situ plasticization not only significantly decreased the wood surface roughness by 34.3% but also endowed the interfacial cell walls with flowability (Figures S3f–j and S4; Table S1). Driven by hot pressing, the plasticized cell walls physically penetrated and intermingled across the adjacent veneer boundaries (Figure 2d,e), causing the originally distinct cell wall interfaces to vanish. Three‐dimensional micro‐computed tomography (Micro‐CT) images further confirmed the formation of a continuous densified structure at the interfacial region (Figures 2f and S5). The physical densification reduced inherent pores and structural defects in the natural wood, creating a mechanically interlocked structure with enhanced micrometer‐scale compatibility. This structurally continuous region may provide a physical pathway for load transfer and impede crack initiation.

#### 2.1.2 Reconstruction and Enhancement of Hydrogen Bond (HB) Network at the Nanoscale and Molecular Scale

The core function of the IL was to effectively disrupt the inherent network of interactions among the cell wall components (cellulose, hemicellulose, and lignin). Temperature‐variable Fourier transform infrared (FT‐IR) spectroscopy and two‐dimensional correlation spectroscopy (2D‐COS) revealed the molecular‐level evolution process (Figure 2g and Figure S6; Table S2). As the temperature increased, the IL weakened the native HB network within the cell wall through new HBs (O–H stretching vibration shifted from 3359 to 3375 cm^−1^). Subsequently, it disrupted the cellulose crystalline regions and interacted with the lignin aromatic skeleton (cellulose–CH_2_ symmetric bending and lignin aromatic skeleton vibrations shifted from 1426 to 1418 cm^−1^). Furthermore, as shown in Figure S7, the IL promoted cleavage of lignin–carbohydrate ester bonds (C═O stretching vibration shifted from 1741 to 1735 cm^−1^, and the intensity of the C–O ester band at 1234 cm^−1^ significantly decreased) [29]. Ultimately, this triggered a partial breakdown of cellulose backbone glycosidic bonds (asymmetric C─O─C stretching vibration shifted from 1173 to 1167 cm^−1^, and the degree of polymerization of cellulose in IPW decreased by 36% relative to that in natural wood) (Figure S8). These results indicated that the IL transformed constrained cell wall components into plasticized structures with increased conformational freedom.

The results of molecular dynamics (MD) simulations further indicated that the HB networks of native lignocellulose (where Cel, Hem, and Lig represent cellulose, hemicellulose, and lignin, respectively, e.g., Cel–Cel, Hem–Hem, Cel–Hem, and Hem–Lig) in the IPW interface (Model B1) were disrupted and replaced by a large number of newly formed biomass–IL HBs (Cel–IL, Hem–IL, and Lig–IL) (Figure S9). This replacement effectively plasticized the interface, leading to expansion of the solvent‐accessible surface area (SASA) (Figure S10). Consequently, hydroxyl groups in the plasticized cell walls at the interface became more accessible for interaction, facilitating the formation of a dense HB network upon structural reorganization. Furthermore, during the evolution from the untreated wood interface (Model A) to Model B1, the system shifted from relatively brittle fracture behavior to a more plastic deformation response. This was accompanied not only by an increase in the calculated tensile strength, but also by the emergence of a yield‐like plateau in the stress–strain curve of Model B1 (Figure 2h–j; Figures S11 and S12; Videos S4 and S5). These results support the critical role of noncovalent interactions and molecular chain mobility in the energy dissipation within the simulated cell‐wall system.

#### 2.1.3 Restructuring and Strengthening of Covalent Bond Networks at the Molecular Scale

In general, the formation of high‐strength interfaces depends on the chemical restructuring of the lignin covalent bond network as well. A quantitative analysis of ball‐milled lignin extracted from natural wood and IPW was performed using two‐dimensional heteronuclear single‐quantum correlation nuclear magnetic resonance (2D HSQC NMR) [30]. The analysis revealed that following cell wall plasticization, the dominant ether bonds (β–O–4) and partial carbon–carbon bonds (β–5) within the native lignin underwent significant cleavage, reducing the relative contents from 79.8% and 12.8% to 62.9% and 5.1%, respectively. The proportion of highly cleavage‐resistant carbon–carbon bonds (β–β) significantly increased to 32.0% (Figure 2k,l and Figure S13). These changes in the relative proportions of bond types indicated depolymerization of the lignin macromolecules. Simultaneously, within the aromatic region, the ratio of syringyl (S) to guaiacyl (G) units increased from 0.7 to 1.2 (Table S3), while the hydrogen signals representing the side chains and aromatic nuclei (such as G5 and G6) considerably attenuated. These dynamic evolutions demonstrated that the G units not only underwent preferential degradation but also actively participated in novel crosslinking reactions via their side chains and aromatic nuclei. Accordingly, the lignin within the interfacial cell walls experienced significant covalent bond cleavage and in situ reconstruction. Ultimately, a covalent network with enhanced mechanical stability was formed [31].

### 2.2 Exceptional Water Resistance and Environmental Durability Enabled by Water‐Induced Regeneration

The long‐term performance stability of biomass‐based composites in humid or aqueous environments is a major factor that determines their applicability as engineering materials. The wet shear strength of IPW lap joints increased from 0.0 MPa (untreated wood) to 1.9 MPa (Figure S14). Even after two rigorous hygrothermal cycles consisting of boiling in water for 4 h and subsequent drying at 63°C for 16 h, the material still exhibited no interfacial delamination. Its dry shear strength decreased by only 3%, and the wood failure rate remained close to 100% (Figure 3a and Figure S15). The mechanical properties of the welded interface were superior to those of previously reported formaldehyde‐ and bio‐based adhesives (Figure 3b) [36, 37, 38, 39, 40]. Additionally, it demonstrated excellent stability against various organic solvents, including formic acid, acetic acid, and acetone (Figure 3c,d), confirming its durability under extreme wet, heat, and chemical conditions.

**FIGURE 3:**
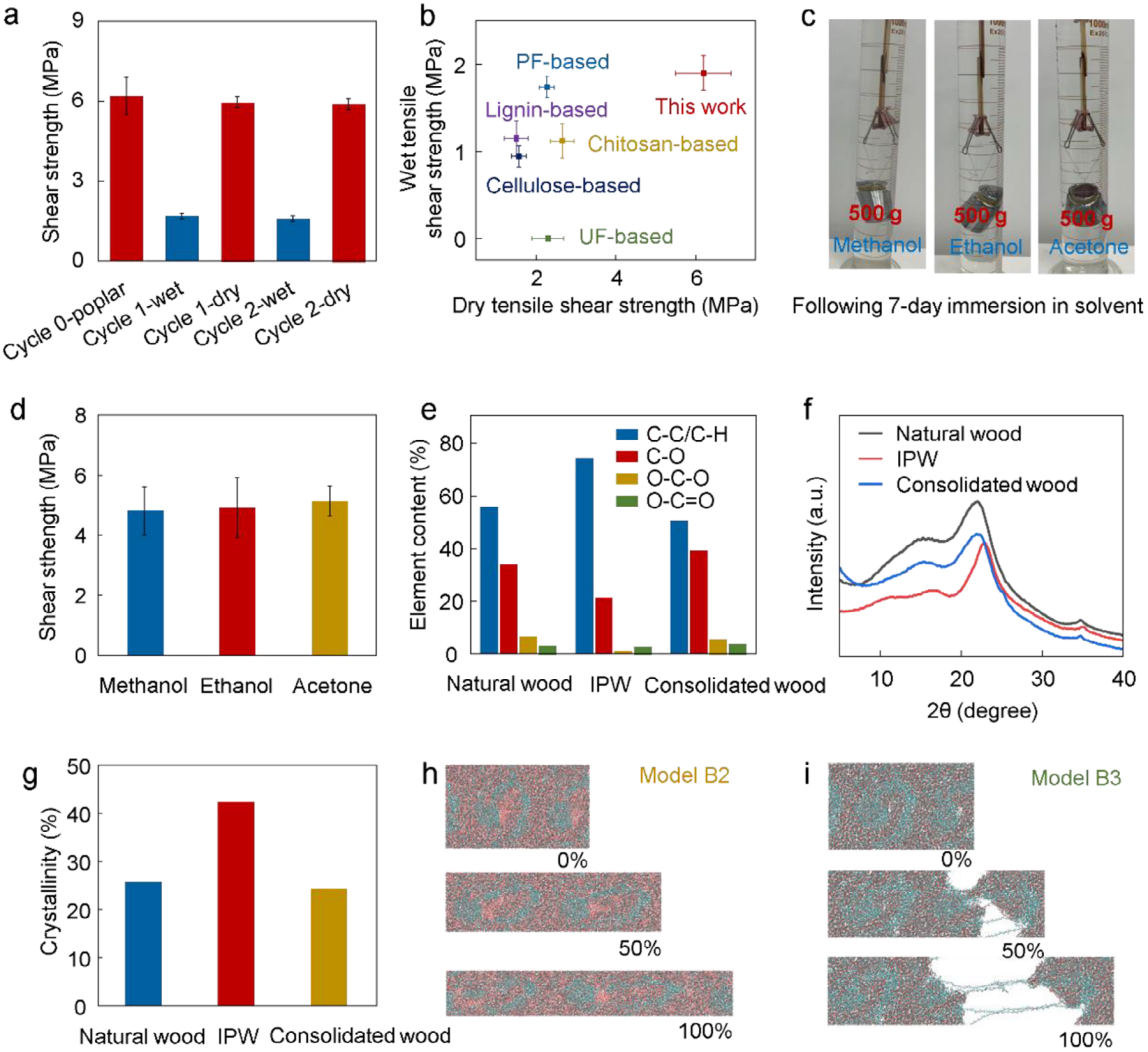
Exceptional water resistance and environmental durability enabled by water‐induced regeneration. (a) Dry and wet shear strengths of IPW lap joints following two rigorous hygrothermal cycles consisting of boiling in water for 4 h and subsequent drying at 63°C for 16 h. (b) Comparison of dry and wet tensile shear strengths of IPW lap joints and wood bonded with other adhesives. (c) Photographs and (d) shear strengths of IPW lap joints following 7 days of immersion in formic acid, acetic acid, and acetone. (e) X‐ray photoelectron spectroscopy (XPS) analysis of carbon functional group contents in natural wood, IPW, and consolidated wood. (f) X‐ray diffraction (XRD) patterns of natural wood, IPW, and consolidated wood. The characteristic peaks of natural wood at 15.3°, 22.1°, and 34.9° correspond to the (110), (200), and (040) lattice planes of cellulose I, respectively [42]. (g) Crystallinity index (CI) of natural wood, IPW, and consolidated wood. The increase in CI from 25.8% to 42.5% following IL plasticization was attributed to the disruption of amorphous lignocellulosic regions. (h, i) Morphological evolution during tensile deformation for (h) Model B2 and (i) Model B3.

The retention of mechanical performance of the welded interface upon contact with water was mainly attributed to its water‐induced regeneration mechanism. As shown in Figure 3e and Figure S16, the relative content of C─C/C─H bonds corresponding to the IL and lignin was lower at the consolidated wood interface than that of natural wood. This confirmed that water molecules effectively displaced the IL from the interfacial region, leading to regeneration of plasticized cell walls (Figure S17). The crystallinity index (CI) of cell walls at the welded interface decreased from 42.5% to 24.4% after regeneration and final consolidation (Figure 3f,g). This indicates that following displacement of the IL by water molecules, the previously preferentially plasticized components solidify at the interface and largely regenerate into a dense amorphous polymer network, thereby providing the material with exceptional water resistance [41].

MD simulations provided visualization of the microscopic evolution of water‐induced regeneration and interfacial performance retention. The evolution from Model B1 to the regenerated wood interface (Model B2) involved the displacement of IL by water molecules, leading to structural contraction of biomass molecular chains and a reduction in the SASA (Figure S10). The subsequent dehydration process promoted the reorganization of the interfacial network, converting HBs between water and biomass into HBs between biomass. This led to the formation of a consolidated wood interface (Model B3) (Figure S9). The ultimate tensile strength of Model B3 was comparable to that of Model B1. This indicated that the interface formed after water‐induced regeneration and final consolidation was independent of the IL, exhibiting an intrinsic cohesive strength comparable to that of the plasticized interface (Figures 2j and 3h,i; Figure S18; Videos S6 and S7). Furthermore, the plasticization temperature of the wood was lower than the initial thermal decomposition temperature of the IL. Meanwhile, the chemical structure of the heat‐treated IL showed no significant changes compared with the fresh one (Figures S19–S21). This indicates that the water‐induced regeneration mechanism may support closed‐loop solvent recovery and sustainable recycling in the future.

### 2.3 IL‐Plasticized Controllable Densification and Potential for High‐Performance Engineering Applications

The in situ plasticization of wood cell walls using IL not only enabled seamless interfacial bonding but also enhanced the density of the material at the macroscopic scale through pressure control. Unidirectional assembly and controlled hot pressing caused a reduction in the thickness of the IPW‑SRSM by approximately 60% and a 2.3‑fold increase in density, from about 0.54 g cm^−3^ (for natural wood) to 1.26 g cm^−3^ (Figure 4a,b and Figure S22a). Moreover, scanning electron microscopy (SEM) analysis showed that as the applied pressure increased from 4.5 to 18.0 MPa, the cell walls transitioned from a disordered state to a highly ordered and dense structure (Figure 4c and Figure S23). However, excessively high pressure (>9.0 MPa) caused cell walls to be crushed, impairing material performance (Figure 4d; Figures S24 and S25). This suggests that the final density and microstructure of the material can be tailored by controlling the pressure, thereby enabling on‐demand performance regulation.

**FIGURE 4:**
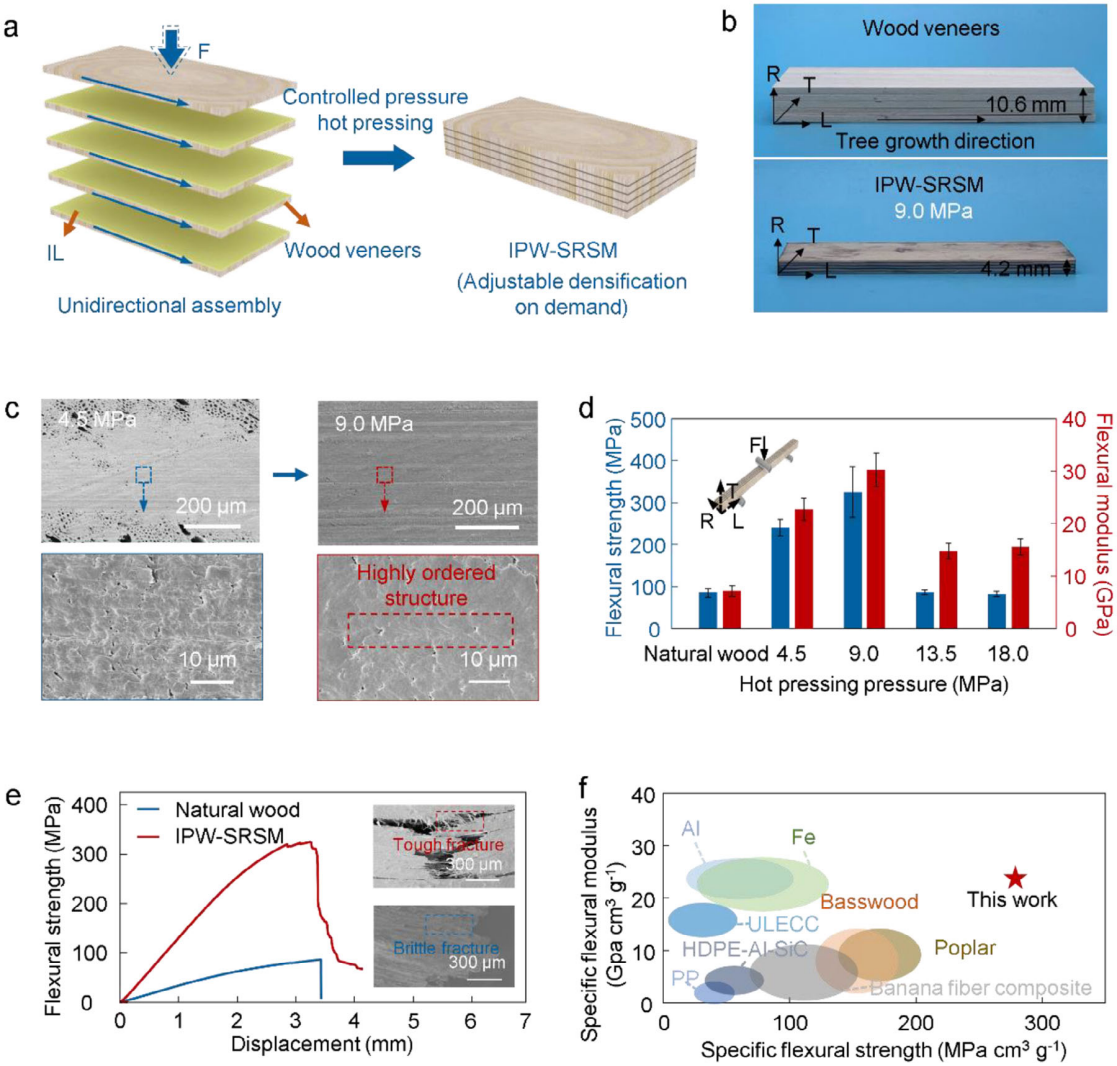
IL‐plasticized controllable densification and potential for high‐performance engineering applications. (a) Schematic illustration of the manufacturing process for IPW‐SRSM. (b) Macroscopic photographs of unbonded wood veneers and IPW‐SRSM. (c) Cross‐sectional SEM images of IPW‐SRSM fabricated under applied pressures of 4.5 and 9.0 MPa. (d) Flexural strength and flexural modulus of natural wood and IPW‐SRSM fabricated under applied pressures of 4.5, 9.0, 13.5, and 18.0 MPa, tested in the transverse (T) direction. (e) Stress–strain curves of natural wood and IPW‑SRSM fabricated under a pressure of 9.0 MPa. (f) Ashby plot comparing the specific flexural strength and specific flexural modulus of IPW‐SRSM with those of metals, conventional building materials, polymer materials, natural wood, and UF‐bonded reconstituted structural material (UF‐RSM).

In a three‐point bending test under transverse (T) loading, the IPW‐SRSM prepared through controlled densification exhibited a flexural strength of 325.3 ± 15.1 MPa and a flexural modulus of 32.2 ± 3.2 GPa, which were 4.1 and 4.5 times those of natural wood, respectively (Figure 4d). Compared with natural wood and UF‐bonded reconstituted structural material (UF‐RSM), IPW‐SRSM demonstrated superior mechanical properties, including flexural strength and modulus under T and radial (R) loading, tensile strength, and impact toughness (Figure S26). Moreover, its impact resistance is comparable to that of previously reported high‐performance wood [43]. The unification of strength and toughness in engineering materials remains a long‐standing challenge [44]. Notably, the stress–strain curve of IPW‐SRSM exhibited tough fracture characteristics (Figure 4e), and its work of fracture reached 34.4 kJ m^−2^, which was approximately 4.5 times that of natural wood (7.8 kJ m^−2^) (Figure S22b). This indicates that the material absorbed a substantial amount of energy through plastic deformation prior to final failure. The fiber bending and microfibril bridging phenomena observed in the SEM image of the fracture surface (Figure S27) further confirmed the high toughness of the material. Furthermore, the water‐soak delamination rate test of samples demonstrated its excellent water resistance (Figure S28). Owing to the inherent lightweight nature of wood, the specific flexural strength (276.2 MPa cm^3^ g^−1^) and specific flexural modulus (24 000 MPa cm^3^ g^−1^) of the IPW‐SRSM were superior to those of typical metals, conventional building materials, polymer materials, and natural wood (Figure 4f and Table S4). The results demonstrate that the IPW‐SRSM holds significant potential in sustainable construction, lightweight automotive components, and aerospace, among others.

## 3 Conclusion

In this study, a simple and sustainable strategy that combined interfacial reconstruction with water‐induced regeneration was developed to construct seamless interlocking interfaces featuring high strength and exceptional resistance to water and solvents. Driven by the synergistic effects of mechanical interlocking, enhanced hydrogen bonding, and a reconstructed covalent network, the initially heterogeneous interfaces transformed into homogeneous ones exhibiting compositional and structural continuity. Consequently, the IPW lap joints achieved a high dry shear strength of 6.2 MPa, which surpassed the bulk strength of natural wood. Notably, water not only functioned as a displacement medium to extract the IL but also drove the secondary reconstruction and performance retention of the interface. This unique mechanism intrinsically endowed the IPW joints with exceptional water stability, enabling them to exhibit a remarkable strength retention rate of 97% even after two extreme boiling water cycles. Furthermore, optimal macroscopic material properties were successfully achieved via precise pressure regulation, yielding outstanding specific flexural strength (276.2 MPa cm^3^ g^−1^) and specific flexural modulus (24 000 MPa cm^3^ g^−1^) that were comparable or even superior to those of certain metals and engineering plastics. Overall, this work demonstrates the significant application potential of high‐performance SRSMs in critical engineering domains such as sustainable construction, automotive lightweighting, and aerospace engineering.

## 4 Experimental Section

### 4.1 Materials

Poplar, birch, pine, and teak wood veneers with nominal thicknesses of 2 and 3 mm were obtained from a local woodworking shop (Shijiazhuang, Hebei Province, China). 1‐Ethyl‐3‐methylimidazolium acetate ([Emim]Ac, 98%), 1‐methylimidazole (≥99%), 1‐ethylimidazole (99%), 1‐butylimidazole (99%), triethyl phosphate (99%), dimethyl phosphite (99%), trimethyl phosphate (98%), copper (II) ethylene diamine solution (CED, 1 m), ethyl acetate (≥99%), dioxane (99.5%), formic acid (99%), acetic acid (99%), ethanol (99.5%), and phloroglucinol (99%) were purchased from Sigma–Aldrich Co., Ltd. Acetone (99%) and hydrochloric acid (AR) were purchased from Heilongjiang Xilong Scientific Co., Ltd (Harbin, Heilongjiang Province, China). The urea‐formaldehyde (UF) adhesive was supplied by Xingang Group (Linyi, Shandong Province, China). All chemicals were used without further purification. Laboratory‐prepared deionized water was utilized for all experimental procedures. 1‐Allyl‐3‐methylimidazolium chloride ([Amim]Cl), 1‐ethyl‐3‐methylimidazolium diethyl phosphate ([Emim]DEP), 1,3‐dimethylimidazolium methyl phosphonate ([Mmim]MP), 1‐ethyl‐3‐methylimidazolium methyl phosphonate ([Emim]MP), and 1‐butyl‐3‐methylimidazolium methyl phosphonate ([Bmim]MP) were synthesized according to the description in the literature [45].

### 4.2 Preparation of Single‐Lap Joints

Two wood veneers (100 × 100 × 3 mm^3^) were partially overlapped parallel to the longitudinal grain direction to form a single‐lap configuration with an overlapping area of 25 × 100 mm^2^. A precisely controlled amount of ionic liquid (IL) (0–1.0 g) was uniformly applied to the overlap region. Subsequently, the assembled veneers were hot pressed at target temperatures ranging from 60°C to 220°C under a pressure of 3.0 MPa for 10 to 60 min. Following hot pressing, the bonded laminates were cooled to room temperature under pressure, and then machined into standard single‐lap shear test samples with a final width of 25 mm. A schematic illustration of the fabrication procedure is provided in Figure S29.

### 4.3 Preparation of IL‑Plasticized Wood Sustainable Reconstituted Structural Material (IPW‑SRSM) and UF‐Bonded Reconstituted Structural Material (UF‐RSM)

Five wood veneers (200 × 100 × 2 mm^3^) were aligned parallel to the longitudinal grain direction. The IL was uniformly brushed onto the mating surfaces of adjacent veneers at a spread rate of 150 g m^−2^. The assembled lay‐ups were subsequently transferred into a hot press. To optimize the interfacial densification, the in situ plasticization and welding process was conducted at 180°C under varying pressures ranging from 4.5 to 18.0 MPa for 30 min. Commercial UF adhesive was applied to the veneer surfaces at the same spread rate, and the assemblies were hot‐pressed at 120°C under a pressure of 9.0 MPa for 30 min. Upon completion, all assemblies were cooled to room temperature under pressure to yield the final IPW‐SRSM and UF‐RSM.

### 4.4 Extraction of Ball‐Milled Lignin From Natural Wood and IPW

Following a previous study [22], ball‐milled lignin was extracted from natural wood and IPW. The detailed procedure was as follows: At 25°C, 20 g of ball‐milled wood powder was extracted twice with 400 mL of aqueous dioxane (96%, v/v), with each extraction lasting for 24 h. The combined filtrates were concentrated by rotary evaporation and freeze‐dried to obtain crude lignin. The crude lignin was subsequently suspended and washed in 20 mL of an acetic acid/water solution (9:1, v/v) to remove soluble impurities. The undissolved precipitate was then collected via centrifugation. This precipitate was washed with deionized water until a neutral pH was achieved and subjected to a second lyophilization step to obtain the final purified ball‐milled lignin.

### 4.5 Morphological Characterization

The [Emim]Ac plasticization process of poplar wood was monitored in real time using an in situ polarized light microscope (DM2700P, Leica, Germany). Transverse wood sections were impregnated with [Emim]Ac and placed in a quartz crucible. The samples were then heated from room temperature to 180°C at a rate of 50°C min^−1^ and held at this temperature while the real‐time morphological changes of the wood were observed. The surface morphology and roughness of the radial sections of both the natural wood and IPW were measured using a laser microscope (VK‐X150, KEYENCE, Japan) with a wavelength of 684 nm. The microscopic morphology of the wood samples was characterized using scanning electron microscopy (SEM, Apreo S, Thermo Scientific, USA). Prior to SEM observation, the non‐conductive samples were sputter‐coated with a thin layer of platinum. To clearly observe the welded region, Wiesner staining was performed on the joint interface sections. Specifically, the sections were first immersed in a 2% phloroglucinol solution in 95% ethanol for 5 min, followed by the addition of 2–3 drops of 6 m hydrochloric acid solution to induce the colorimetric reaction. This stain reacted specifically with the coniferyl aldehyde groups naturally present in the native lignin, imparting a distinct purple color to the non‐welded wood. In contrast, the structurally modified lignin in the welded region remained unstained. The morphology of the lap joint interface was then observed using an optical microscope (DM1000, Leica, Germany). Furthermore, the internal three‐dimensional microstructures of the welded materials were reconstructed using micro‐computed tomography (micro‐CT, SKYSCAN1272, Bruker, Germany).

### 4.6 Structural Characterization

Temperature‐variable Fourier transform infrared (FT‐IR) spectra were acquired using a Bruker EQUINOX55 spectrometer. Two‐dimensional correlation spectroscopy (2D‐COS) synchronous and asynchronous maps were generated from the spectral data using 2D Shige software (version 1.3; Shigeaki Morita, Kwansei Gakuin University, Japan, 2004–2005). The characteristic functional groups of the wood samples were identified using FT‐IR (Tensor II, Bruker, USA) spectroscopy in the range of 4000–400 cm^−^^1^. 2D HSQC NMR measurements were conducted using a Bruker AVANCE III HD 600 MHz spectrometer. To prepare the samples, 80 mg of ball‐milled lignin was dissolved in 0.6 mL of DMSO‐d_6_. Semi‐quantitative analysis was then performed to determine the relative abundances of lignin substructures and the S/G ratio, following previously established procedures. The relative proportions of the side‐chain linkages were calculated according to the formula A_α_ + B_α_ + C_α_ = 100%, where A_α_, B_α_, and C_α_ correspond to the integrals at the α‐position for β–O–4′ (A), β–5′ (B), and β–β′ (C) linkages. All integrations were performed under identical conditions. The crystalline structure of the wood samples was characterized by X‐ray diffraction (XRD, Smart Lab SE, Rigaku, Japan) with a scanning range of 5°–60° and a scanning rate of 10° min^−1^. X‐ray photoelectron spectroscopy (XPS) analysis was performed using an ESCALAB 250 instrument (Thermo‐Fisher Scientific, USA), and all binding energies were calibrated with respect to the adventitious C 1s peak at 284.8 eV. The thermal stability of the samples was evaluated using a simultaneous thermal analyzer (TGA2, Mettler Toledo, USA) by heating from 30°C to 800°C at a rate of 10°C min^−1^ under a nitrogen atmosphere with a flow rate of 50 mL min^−1^. Thermal analysis of the wood samples was conducted under a nitrogen atmosphere using a differential scanning calorimeter (DSC, Q2000, TA, USA). The samples were heated from room temperature to 200°C at a rate of 10°C min^−1^.

### 4.7 Mechanical Tests

The mechanical properties of single‐lap joint samples, natural wood, and IPW‐SRSM were tested using a universal testing machine (AGS‐X, Shimadzu, Japan). The single‐lap joint samples had overall dimensions of 175 × 25 mm^2^, with an overlap area of 25 × 25 mm^2^. The tensile samples of natural wood and IPW‐SRSM measured approximately 100 × 5 × *h* mm^3^ (where *h* represents the specimen thickness). Both types of samples were clamped at both ends and stretched along their length until failure at a constant test speed of 5 mm min^−1^. The bending samples measured approximately 100 × *h* × *h* mm^3^. These samples were subjected to three‐point bending tests with a span of 20 *h* mm between the two bottom rollers, while the top roller was pressed downward at the center at a speed of 5 mm min^−1^. The impact toughness of samples was evaluated using a pendulum impact testing machine (JK‐XBL‐FS, Shanghai Jiangkai Machinery Co., Ltd., China). Rectangular samples with dimensions of 100 × 10 × *h* mm^3^ were prepared. The impact test was conducted with a span of 36 mm, and the pendulum was released to strike the sample flatwise. For the aforementioned mechanical tests, dry‐state samples were naturally cooled to room temperature (approximately 25°C) and tested immediately. For wet‐state tests, the samples were immersed in water at 25°C for 10 min following the corresponding hydrothermal treatments, and tested within 30 min after their removal. Three independent samples were evaluated for each mechanical test. All reported data represent the average values, with error bars indicating the standard deviation.

### 4.8 Solvent‐Resistance Tests

The IPW lap‐joint samples were immersed separately in formic acid (99%), acetic acid (99%), and acetone (99%) at 25°C for 7 days. After immersion, the samples were removed and dried at 70°C for 3 h, followed immediately by mechanical testing. Three replicate samples were evaluated for each treatment condition, and the average values were calculated.

### 4.9 Measurement of Crystallinity Index (CI)

The CI of the wood samples was calculated from the XRD patterns using the peak‐height method according to Equation (1):

(1) $$\mathrm{CI}=\frac{I_{200}-I_{\mathrm{am}}}{I_{200}}\;\times 100\%$$

where *I*_200_ is the maximum intensity of the (200) lattice plane diffraction peak, and *I*_am_ is the minimum intensity between the (200) and (110) peaks.

### 4.10 Density Measurement of Wood Samples

The density of the wood samples was calculated using Equation (2).

(2) $$\rho =\frac{m}{V}$$

where *ρ* represents the density of the sample, *m* is the mass of the sample, and *V* is the volume of the sample.

### 4.11 Water‐Soak Delamination Rate Test of IPW‑SRSM and UF‐RSM

The samples (75 × 75 × *h* mm^3^) were fully immersed in boiling water for 4 h, then transferred to deionized water at 10–25°C for 1 h. Afterward, the samples were dried in an oven at (70 ± 3)°C until their mass reached 100%–110% of the initial mass. This procedure was repeated twice. The delamination rate of samples was calculated according to Equation (3).

(3) $$D=\frac{l_{1}}{l_{2}}$$

where *D* represents the delamination rate of samples, *l*_1_ is the length of the cracked adhesive layer in the samples, and *l*_2_ is the total length of the adhesive layer in the samples.

### 4.12 Molecular Dynamics (MD) Simulation Section

#### 4.12.1 Model Construction and Simulation Systems

In this study, four simulation systems were constructed and investigated based on the plant cell wall model previously reported [33]. Starting from the original model, three derivative systems were generated by systematically inserting and removing solvent molecules, with the aim of elucidating the effects of different solvent environments and solvent removal processes on the structure and properties of the cell wall.

Model A (untreated wood interface) served as the reference system. It directly adopted the published initial coordinates of the native cell wall composite without any solvation treatment, representing the dry‐state structure of the pristine cell wall.

Model B1 (IL‐plasticized wood interface) was designed to simulate the state in which the cell wall was fully infiltrated by [Emim]Ac. This model was constructed on the basis of Model A by introducing [Emim]Ac into the cell wall pores using an iterative insertion approach. Specifically, one [Emim]Ac ion pair was inserted into the system at each step, followed by energy minimization and a short 5 ps NPT relaxation to alleviate local steric clashes. This “insertion‐minimization‐relaxation” cycle was repeated until the total mass of the ionic liquid reached 100% of the dry mass of the cell wall.

Model B2 (regenerated wood interface) was employed to simulate the hydrated regenerated cell wall structure after IL treatment. In this procedure, all IL molecules were first removed from Model B1, after which water molecules were randomly inserted into the pores in a single step using the insert‐molecules tool in GROMACS, until the total water mass reached 30% of the dry mass of the cell wall.

Model B3 (consolidated wood interface) was used to represent the dried state after hydration. This model was directly obtained by deleting all water molecules from the equilibrated Model B2.

After construction, all systems were sequentially subjected to energy minimization, short NPT relaxation, and long‐term production simulations.

#### 4.12.2 Force Field and Simulation Parameters

All components were described using the GROMOS 54A7 force field, consistent with previous studies. The topology parameters of the [Emim]Ac were generated using the Automated Topology Builder (ATB; https://atb.uq.edu.au) online server and exported in the GROMOS 54A7 format to ensure force‐field consistency across the system. Water molecules were modeled using the SPC/E model compatible with the GROMOS 54A7 force field.

#### 4.12.3 Energy Minimization

After system construction, all models were first subjected to energy minimization using the steepest descent algorithm. The convergence criterion for the maximum force was set to 500 kJ mol^−1^ nm^−1^, with a maximum of 5000 steps, in order to eliminate unfavorable atomic contacts.

#### 4.12.4 System Equilibration

Prior to production simulations, each system underwent a systematic hot pressing and equilibration protocol. Initially, a 500 ps NPT simulation was performed at 400 K and 1000 bar using the V‐rescale thermostat and the Berendsen barostat to eliminate initial vacuum regions. Subsequently, a second 500 ps NPT equilibration was carried out at 300 K and 1 bar to allow the system to fully relax to the target thermodynamic state.

#### 4.12.5 Production Simulation

After confirming the absence of apparent vacuum regions and the stabilization of system energy, a 10 ns NPT production simulation was conducted. The thermodynamic conditions were maintained using the V‐rescale thermostat (300 K) and the Parrinello‐Rahman barostat (1 bar). An integration time step of 1 fs was employed. Covalent bonds were constrained using the LINCS algorithm. Nonbonded interactions were handled using a Verlet neighbor list, with cutoff distances of 1.2 nm for both short‐range Coulombic and van der Waals interactions. Long‐range electrostatic interactions were treated using the particle mesh Ewald (PME) method, and van der Waals interactions beyond the cutoff were corrected using energy and pressure tail corrections (DispCorr = EnerPres).

#### 4.12.6 Tensile Simulations

To evaluate the effects of different treatments on the macroscopic mechanical properties of the cell wall, uniaxial tensile simulations were performed on four fully relaxed systems (Models A, B1, B2, and B3). Each system was first periodically replicated along the tensile direction (*x*‐axis) to construct an extended model, followed by a 500 ps NPT equilibration at 300 K and 1 bar to ensure sufficient interfacial relaxation. Tensile simulations were then conducted under the NPT ensemble at 300 K, with the pressure along the tensile direction (*x*‐axis) set to 0, while the transverse pressures (*y‐* and *z‐*axes) were maintained at 1 bar, allowing for free lateral deformation. During the simulations, the system was stretched along the *x*‐axis at a constant strain rate until a total strain of 100% was reached, and the corresponding stress–strain responses were recorded for subsequent mechanical analysis.

## Author Contributions

**Huizheng Wu**: Writing – original draft, Writing – review & editing, data curation, investigation. **Yi Kong**: Formal analysis, Software, Writing – review & editing. **Liangxian Liu**: Data curation. **Yuyan Jiang**: Data curation. **Jiahuan He**: Investigation. **Haiyu Li**: Data curation. **Ming Wei**: Data curation. **Dexiu Min**: Data curation. **Jian Li**: Writing – review & editing, Conceptualization. **Yanjun Xie**: Writing – review & editing, Investigation. **Chi Zhang**: Supervision, Software, Funding acquisition, Writing – review & editing. **Shaoliang Xiao**: Supervision, Funding acquisition, Writing – review & editing, Conceptualization, Project administration.

## Conflicts of Interest

The authors declare no conflicts of interest.

## Supporting information




**Supporting File 1**: advs78399‐sup‐0001‐SuppMat.docx.

**Supporting File 2**: advs78399‐sup‐0002‐VideoS1.mp4.

**Supporting File 3**: advs78399‐sup‐0003‐VideoS2.mp4.

**Supporting File 4**: advs78399‐sup‐0004‐VideoS3.mp4.

**Supporting File 5**: advs78399‐sup‐0005‐VideoS4.mp4.

**Supporting File 6**: advs78399‐sup‐0006‐VideoS5.mp4.

**Supporting File 7**: advs78399‐sup‐0007‐VideoS6.mp4.

**Supporting File 8**: advs78399‐sup‐0008‐VideoS7.mp4.

## Data Availability

The data that support the findings of this study are available from the corresponding author upon reasonable request.
