# Supplementary material for: Multiscale Interfacial Engineering Enables Robust Reconstituted Wood
Source: Adv Sci (Weinh). 2026 Oct 28:e78399. Online ahead of print. doi: 10.1002/advs.78399 (PMC13715676; doi:10.1002/advs.78399)
Supplement: Supplementary file 1 [file ADVS-9999-e78399-s007.docx]

**Supplementary Information**

*Huizheng Wu^1#^, Yi Kong^4,5#^, Liangxian Liu^1^, Yuyan Jiang^1^, Jiahuan He^1^, Haiyu Li^1^, Ming Wei^1,3^, Dexiu Min^3^, Jian Li ^2^, Yanjun Xie^1^, Chi Zhang^*4,5^, Shaoliang Xiao^*1^*

1. Key Laboratory of Bio-based Material Science & Technology (Ministry of Education), Northeast Forestry University, Harbin, PR China

2. Engineering Research Center of Advanced Wooden Materials (Ministry of Education), Northeast Forestry University, Harbin, PR China

3. Shandong Xingang Enterprise Group Co., Ltd, Shandong, PR China

4. School of Flexible Electronics (SoFE), Sun Yat-sen University. Shenzhen 518000.China

5. State Key Laboratory of Optoelectronic Materials and Technologies (OEMT), Sun Yat-sen University, Guangzhou 510275, China

^#^ These two authors contributed equally to this work.

^*^ Email: [shaoliangxiao@nefu.edu.cn](mailto:shaoliangxiao@nefu.edu.cn), [zhangchi89@mail.sysu.edu.cn](mailto:zhangchi89@mail.sysu.edu.cn)

**Note 1:** The mechanical performance of IL‑plasticized wood (IPW) lap joints is synergistically governed by the hot pressing temperature, time, and IL mass. Appropriately increasing the temperature and extending the hot pressing time can effectively promote interfacial plasticization, thereby significantly enhancing the mechanical properties. However, temperatures exceeding 180 °C, durations beyond 30 min, or prolonged exposure to high temperatures trigger the thermal degradation and embrittlement of wood components, resulting in a deterioration of mechanical strength. Moreover, increasing the IL mass to 0.75 g provided an appropriate welding depth for sufficient IL penetration and cell-wall mechanical interlocking. This effectively promoted the plasticization of the wood surface and constructed a molecular entanglement network at the welding interface, enabling the dry and wet tensile shear strengths of the IPW lap joints to increase from 0.5 MPa and 0.0 MPa to 6.2 MPa and 1.9 MPa, respectively. Nevertheless, when the IL mass was further increased to 1.0 g, the excessive welding depth induced over-plasticization of the wood, causing both strengths to decrease to 5.8 MPa and 1.8 MPa, respectively.

**Figure S1.** (a) The wet shear strength of IPW lap joints prepared using different ionic liquids (ILs). (b) The dry/wet shear strength of IPW lap joints prepared using different wood species.

**Figure S2.** (a) Dry and (b) wet tensile shear strengths of IPW lap joints prepared under different hot pressing times/temperatures. (c) Dry and wet tensile shear strengths of IPW lap joints prepared under different hot pressing times (10–60 min). (d) Dry and wet tensile shear strengths and welding depths of IPW lap joints prepared with different IL masses. (e) Dry and wet tensile shear strengths and (f) failure mode images of IPW lap joints after storage at 180 °C for 24 h. (Note: The wet tensile shear strength was tested after soaking the samples in water at 63 °C for 3 h.).

**Figure S3.** (a) Schematic diagram of wood transverse section. Polarized light microscopy images of wood (b) after impregnation with IL and (c) after plasticization. SEM images of transverse section for (d) natural wood and (e) IPW. (f) Schematic diagram of wood radial section. Laser microscopy images of radial sections for (g, i) natural wood and (h, j) IPW.

**Figure S4.** Variation in surface roughness on the radial sections of IPW compared to natural wood.

**Figure S5.** (a, b) Micro-computed tomography three-dimensional reconstruction of the IPW lap joint.

**Figure S6.** (a) Synchronous and (b) asynchronous 2D-COS spectra derived from the Variable‐temperature FT-IR data.

**Figure S7.** FT-IR spectra of natural wood and consolidated wood, recorded in the 1100–1800 cm^−1^ region.

**Figure S8.** Degree of polymerization (DP) of cellulose in natural wood and IPW.

**Figure S9.** Number of HBs between components in Model A, B1, B2, and B3.

**Figure S10.** Solvent-accessible surface area (SASA) of components in Model A, B1, B2, and B3.

**Figure S11.** Morphological evolution during tensile deformation for (a) Model A and (b) Model B1.

**Figure S12.** Evolution of SASA as a function of strain for Model (a) A, (b) B1, (c) B2, and (d) B3. Over a strain range of 10% to 100%, a pronounced yield plateau without fracture was exhibited by Model B1. The decrease in total biomass SASA in the 50% to 100% strain regime was mainly associated with cellulose rearrangement.

**Figure S13.** Substructure and units of lignin.

A: β–aryl ether substructures (β–O–4); B: resinol (β–β) substructures; C: phenylcoumaran substructures (β–5); S/S’: Syringyl; G/G’: Guaiacyl

**Figure S14.** Wet tensile shear strength of natural wood and IPW lap joint after immersion in water at 63 °C for 3 h.

**Figure S15.** Failure mode analysis of IPW lap joints.

**Figure S16.** XPS C1s spectra of (a) natural wood, (b) IPW, and (c) consolidated wood.

**Figure S17.** Transverse sectional SEM morphology of natural wood, IPW, and consolidated wood.

**Figure S18.** Morphological evolution during tensile deformation for (a) Model B2 and (b) Model B3.

**Figure S19.** DSC profiles of natural wood and IPW samples.

**Figure S20.** (a) TGA and (b) derivative weight curves of [Emim]Ac.

**Figure S21.** FT-IR spectra of fresh [Emim]Ac and [Emim]Ac thermally treated at 180 °C for 30 min.

**Figure S22.** (a) Density and (b) work of fracture of natural wood and IPW-SRSM.

**Figure S23.** Transverse section SEM images of the IPW-SRSM fabricated under applied pressures of (a, b) 13.5 MPa and (c, d) 18.0 MPa.

**Figure S24.** Tensile strength of natural wood and IPW-SRSM prepared at hot pressing pressures of 4.5, 9.0, 13.5, and 18.0 MPa.

**Figure S25.** Flexural strength and flexural modulus of natural wood and IPW-SRSM fabricated under applied pressures of 4.5, 9.0, 13.5, and 18.0 MPa, tested in the R direction.

**Figure S26.** Mechanical comparisons among natural wood, UF-RSM, and IPW-SRSM. Flexural strength and modulus loaded along the (a) T and (b) R directions. (c) tensile strength. (d) impact toughness.

**Figure S27.** Compared to the smooth fracture surfaces of (a) natural wood and (b) IPW-SRSM exhibits numerous micro-fiber bridges during the fracture process.

**Figure S28.** (a) Comparison of water-soak delamination rates between UF-RSM and IPW-SRSM.

**Figure S29.** Schematic illustrating the preparation of the lap joint.

**Table S1.** Sa and Str values for natural wood and IPW.

|  | Natural wood | IPW |
| --- | --- | --- |
| Sa | 41.701 | 27.411 |
| Str | 0.071 | 0.336 |

**Table S2.** Results of the multiplication of the signs of each cross-peak in 2D COS synchronous and asynchronous spectra of activated wood.

| 1173 | + | + | + |  |
| --- | --- | --- | --- | --- |
| 1426 | + | – |  |  |
| 1741 | + |  |  |  |
| 3359 |  |  |  |  |
|  | 3359 | 1741 | 1426 | 1173 |

According to Noda’s rule, if the multiplication of the cross peaks v1 and v2 (where v1 > v2) in 2D COS synchronous and asynchronous spectra is positive, then v1→v2, indicating that the change in the v1 peak responds earlier than the change in the v2 peak, and vice versa [1].

**Table S3.** Relative content of S and G units in natural lignin and IPW lignin.

|  | S (%) | G (%) |
| --- | --- | --- |
| Natural lignin | 41.6 | 58.4 |
| IPW lignin | 54.0 | 46.0 |

**Table S4.** Comparative performance of materials in density and specific flexural strength.

| Materials | specific flexural strength (Mpa cm^3^ g^−1^) | Specific modulus  (Gpa cm^3^ g^−1^) | Reference |
| --- | --- | --- | --- |
| Al | 55.6 | 25.9 |  |
| Fe | 51.0 | 26.8 |  |
| PP | 41.1 | 1.3 | [2] |
| Banana fiber composite | 56.8–107.6 | 2.3–4.4 | [3] |
| HDPE-Al-SiC | 11.1–54.5 | 0.7–3 | [4] |
| ULECC | 17.5–23.2 | 11.6–15.4 | [5] |
| Natural poplar | 191.1 | 9.9 | [6] |
| Natural basswood | 172.9 | 9.4 | [7] |
| This work | 276.2 | 24.5 |  |

References

1.	X. Shi, P. Wu, "A Smart Patch with On-Demand Detachable Adhesion for Bioelectronics," *Small* 17, no. 26 (2021): 2101220, <http://dx.doi.org/10.1002/smll.202101220>.

2.	R. Nonato, B. Bonse, "A study of PP/PET composites: Factorial design, mechanical and thermal properties," *Polymer Testing* 56 (2016): 167–173, <http://dx.doi.org/10.1016/j.polymertesting.2016.10.005>.

3.	V. Santhanam, R. Dhanaraj, M. Chandrasekaran, N. Venkateshwaran, S. Baskar, "Experimental investigation on the mechanical properties of woven hybrid fiber reinforced epoxy composite," *Materials Today: Proceedings* 37 (2021): 1850–1853, <http://dx.doi.org/10.1016/j.matpr.2020.07.444>.

4.	S. Rana, M. Hasan, M. R. K. Sheikh, A. N. Faruqui, "Mechanical and morphological properties of high density polyethylene-Al-SiC hybrid composites," *Advances in Materials and Processing Technologies* 8, no. 1 (2022): 1135–1146, <http://dx.doi.org/10.1080/2374068X.2020.1853497>.

5.	B. Deng, L. Li, D. Tan, M. Uddin, Z. Cai, K. Yu, "Sustainable and cost-effective ultra-lightweight engineered cementitious composite: Design and material characterization," *Cement & Concrete Composites* 136 (2023): 104895, <http://dx.doi.org/10.1016/j.cemconcomp.2022.104895>.

6.	J. Wang, R. Simson, R. Gleisner, S. Fishwild, M. Begel, J. Zhu, "Carbonate pre-treatment of wood for transformative structural applications through densification," *Industrial Crops and Products* 188 (2022): 115645, <http://dx.doi.org/10.1016/j.indcrop.2022.115645>.

7.	D. Huang, J. Li, S. Li, J. Hu, Z. Cao, Y. Guo, Y. Ding, M. Zhu, Y. Chen, "Self-densified super-strong wood," *Journal of Bioresources and Bioproducts* 10, no. 2 (2025): 199–208, <http://dx.doi.org/10.1016/j.jobab.2025.03.001>.
